# Whole-Exome Sequencing Identified *CFTR* Variants in Two Consanguineous Families in China

**DOI:** 10.3389/fgene.2021.631221

**Published:** 2021-07-02

**Authors:** Binyi Yang, Cheng Lei, Danhui Yang, Zhiping Tan, Ting Guo, Hong Luo

**Affiliations:** ^1^Department of Pulmonary and Critical Care Medicine, The Second Xiangya Hospital, Central South University, Changsha, China; ^2^Research Unit of Respiratory Disease, Central South University, Changsha, China; ^3^Hunan Diagnosis and Treatment Center of Respiratory Disease, Changsha, China; ^4^Department of Cardiovascular Surgery, Clinical Center for Gene Diagnosis and Therapy, The Second Xiangya Hospital of Central South University, Changsha, China

**Keywords:** cystic fibrosis, *CFTR*, bronchiectasis, Chinese, genetic variants, phenotype

## Abstract

**Background:**

Cystic fibrosis (CF) is an autosomal recessive disease caused by genetic variants of the cystic fibrosis transmembrane conductance regulator (*CFTR*) gene. It is a common hereditary disease in Caucasians while rare in the Chinese. Until now, only 87 Chinese patients have been reported with molecular confirmations. The variant spectrum and clinical features of Chinese CF patients are obviously different from those of Caucasians.

**Materials and Methods:**

Whole-exome sequencing was applied to analyze the exome of three individuals who have only the typical CF phenotype in the respiratory system from two consanguineous families. The protein domain and structure analysis were applied to predict the impact of the variants. Sanger sequencing was applied to validate the candidate variants.

**Results:**

A previously reported homozygous variant in *CFTR* (NM_000492.4: c.1000C > T, p.R334W) was identified in proband I. A novel homozygous variant in a polymorphic position (NM_000492.4: c.1409T > A, p.V470E) was identified in two individuals in the family II. The novel *CFTR* variant predicted to be disease-causing is the first, to the best of our knowledge, to be reported in *CFTR*. However, *in vitro* validation is still needed.

**Conclusion:**

Our finding expands the variant spectrum of *CFTR*, reveals clearer clinical phenotype distinction and variant spectrum distinction between Chinese and Caucasian CF patients, and contributes to a more rapid genetic diagnosis and future genetic counseling.

## Introduction

Cystic fibrosis (CF), first reported in 1938, is an inherited autosomal recessive disease involving multiple organs, including the respiratory, digestive, and reproductive systems (Andersen, 1938). Respiratory failure after bronchiectasis and infection is now the main cause of death in CF patients. CF is a common hereditary disease in Caucasians but is rare among Asians, especially the Chinese. The estimated incidence of the disease in Caucasian population is about 1/3,000, but no more than 100 cases that have been reported in China (Liu et al., 2020). The disease is mainly detected through abnormally high chloride concentrations in sweat and gene sequencing.

Cystic fibrosis is caused by variants of the cystic fibrosis trans-membrane conductance regulator (*CFTR*) gene. CFTR is a chloride and bicarbonate transport protein, encoded by the *CFTR* gene located at the long arm of chromosome 7, which has 27 exons. As *CFTR* is mainly expressed in various glandular tissues, the variants can cause abnormal ion transport and secretory function, subsequently leading to bronchiectasis, pancreatic insufficiency, and fertility dysfunction in males (Ratjen et al., 2015). More than 2,000 different variants have been identified in *CFTR*, and the most recent update from the CFTR2 database^1^ recorded a total of 442 variants including 360 CF-causing variants. All variants are divided into six classes according to their mechanisms and pathogenicity. The most prevalent variant in Caucasians, p.F508del, was classified in Class II(Ratjen et al., 2015).

Studies have shown that the clinical phenotypes and variant spectrum of CF patients in China and those of the Caucasian population are very different (Guo et al., 2018). There are approximately 90 Chinese CF patients who have been reported up to now. In recent years, however, as the understanding of CF has deepened and the methods of diagnosis have improved, a growing number of CF patients have been diagnosed and reported in China. Here, we used whole-exome sequencing to analyze the exome of three individuals who have only a typical respiratory system CF phenotype, from two consanguineous families, and found *CFTR* variants including a novel variant in a polymorphic position.

## Materials and Methods

### Ethical Compliance

This study was approved by the Review Board of the Second Xiangya Hospital of Central South University in China. Three patients diagnosed with bronchiectasis from two Chinese consanguineous families participated in the study. Written informed consent was obtained from all participants.

### Whole-Exome Sequencing and Variants Filtering

Whole-exome sequencing was performed on three individuals from two consanguineous families. Peripheral blood samples (3–5 ml) from proband I in family I; proband IIa and his brother IIb in family II were obtained with informed consent, respectively. The genomic DNA was extracted from peripheral blood cells by DNeasy blood and tissue kit (QIAGEN # 69506). The exomes of the probands were captured by Agilent Sureselect Human All Exons V6 kit (Agilent, California, United States) and sequencing on Illumina Hiseq X-10 platform (Illumina Inc, San Diego, United States). The single-nucleotide variants (SNVs) and short insertions and deletions (INDELs) were filtered as follow as we describe (Supplementary Figure 1): (1) Variants in the 1,000 Genomes Project (1,000G^2^), NHLBI-ESP project^3^, and Exome Aggregation Consortium (ExAC^4^) with minor allele frequency (MAF) ≤ 0.01 were included. (2) Intergenic, intronic, and untranslated regions and synonymous variants were excluded from subsequent analyses. (3) Bioinformatics analyses (Sift, Polyphen-2, Mutationtaster, MutationAssessor) were used for the remaining variants. (4) The remaining data were flittered by bronchiectasis-related diseases, which include primary ciliary dyskinesia (PCD), cystic fibrosis, and primary immunodeficiency. (5) Runs of homozygosity (ROH) analysis was also performed due to the fact that all probands came from consanguineous families. The ROH analysis was based on the data from whole-exome sequencing data using the Automap algorithm (Quinodoz et al., 2021).

### Variant Validation With Sanger Sequencing

Sanger sequencing was used to validate the candidate variants found in whole-exome sequencing. Primers for PCR amplification of fragments with individual variants were designed by Primer Premier 5 software (Premier Biosoft, Palo Alto, CA, United States). The sequences of the primers are listed in Supplementary Table 1.

## Results

### Clinical Summary

#### Family 1

**FIGURE 1:**
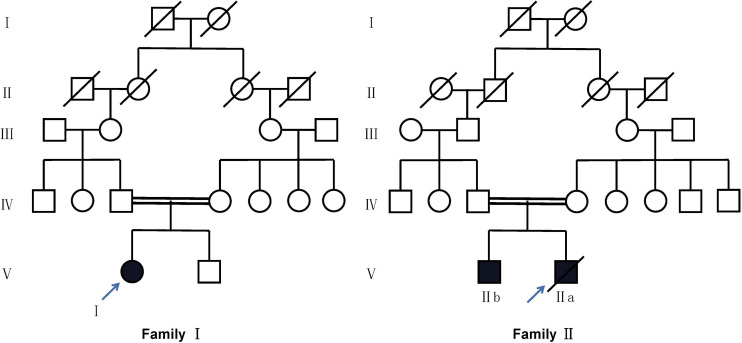
Pedigree of two families with *CFTR* variants. Circles refer to females. Squares refer to male subjects. Solid symbols refer to affected subjects. Crossed-out symbols refer to subjects who have passed away. The arrows indicate the probands.

**FIGURE 2:**
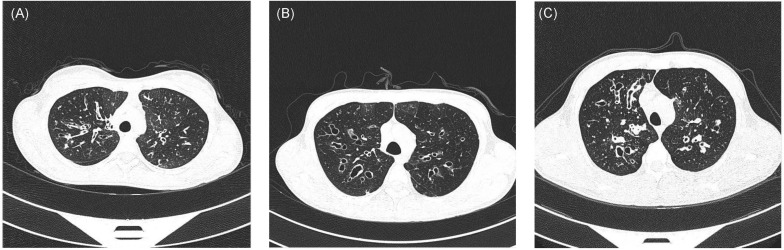
High resolution computed tomography reveals signs of bronchiectasis in all probands [**(A)**, proband I; **(B)**, proband IIa; **(C)**, patient IIb].

Proband I was a 14-year-old girl whose parents are consanguineous (Figure 1). She had suffered from recurrent coughing and expectoration for 2 years and came to our clinic for 1 day due to acute hemoptysis. Physical examination showed moist rales in both lower lungs. CT (computed tomography) showed inflammation in bilateral maxillary sinus, ethmoid sinus, and sphenoid sinus, bronchiectasis in both lungs, and calcification of the liver (Figure 2A). Her nasal nitric oxide concentration (nNO) was 74 parts per billion (ppb). The pulmonary function test indicated that she had severe mixed pulmonary ventilation dysfunction (FEV1% predicted: 48.0%, FEV1/FVC: 80.5%), and the bronchial dilation test was negative. The sputum culture showed infection with *Pseudomonas aeruginosa*. After hemostasis, antibiotic, and expectorant treatment, her symptoms were relieved, and the patient was discharged.

#### Family 2

Proband IIa was a 17-year-old teenager whose parents are also consanguineous (Figure 1). He had been coughing and expectoration repeatedly for 6 years and experiencing shortness of breath for 1 year, and he was admitted to the Respiratory Intensive Care Unit due to suddenly worsening symptoms. Physical examination revealed a barrel chest, some moist rales, and finger clubbing. Arterial blood gas analysis suggested hypercapnic acidosis. Chest radiography suggested bronchiectasis and infection. Color Doppler echocardiography showed mild mitral regurgitation. CT showed chronic inflammation of bilateral maxillary sinuses, an undeveloped sphenoid sinus, and left frontal sinus, along with diffuse bronchiectasis with patchy infection foci (Figure 2B). His nasal nitric oxide concentration (nNO) was 159 ppb. The patient was given ventilator and phlegm treatment. The sputum culture was positive for *Pseudomonas aeruginosa*. The cough and expectoration have not improved significantly after changing antibiotics several times. After sputum aspiration under fiberoptic bronchoscopy, the patient’s coughing and expectoration decreased, and his condition improved significantly. Soon after, the patient was discharged from the hospital.

IIb is the 5-year-old brother of IIa. His nitric oxide concentration (nNO) was 113 ppb. He had no symptoms except for digital clubbing and the CT suggested sinusitis, bronchiectasis, and calcification of the liver (Figure 2C).

### Whole-Exome Sequencing Identified Two Variants in *CFTR*

The whole-exome sequencing was performed on three individuals, and the results were listed in Supplementary Table 2. Notably, 14.24, 7.09, and 7.25 Gb sequencing data were generated, and more than 99% of the targeted regions were covered, with a depth of more than 10×. Non-synonymous variants occurring in exons or splice sites (splicing junction 10 bp) were selected and analyzed after alignment. After the selection of rare variations (minor allele frequency <1%) related to bronchiectasis, one homozygous variant of cystic fibrosis transmembrane conductance regulator (*CFTR*) was identified in each family. The results of our ROH analysis confirmed the homozygous region in chromosome 7 contains the variants of *CFTR*. The detailed results of ROH analysis are shown in Supplementary Figure 2.

A homozygous variant of *CFTR*, c.1000C > T, p.R334W, which had been reported to be disease-causing, was found in proband I(Sheppard et al., 1993).

**FIGURE 3:**
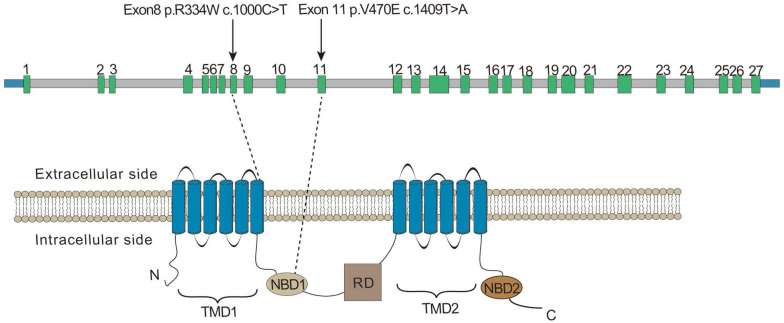
Exon structure of the *CFTR* gene. Domain structure of the CFTR protein. The positions of variant c.1000C > T, p.R334W, and c.1409T > A, p.V470E. TMD, transmembrane domain; NBD, nucleotide binding domain; RD, regulatory (R) domain.

**FIGURE 4:**
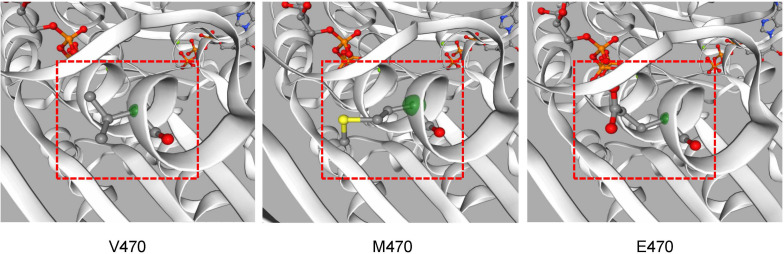
The CFTR protein tertiary structure was predicted by Swiss-model software. Different structures caused by amino acid changes are shown in position 470 of CFTR.

A novel variant of *CFTR*, c.1409T > A, p.V470E was found in proband IIa and IIb. It hadn’t been reported before and hadn’t been recorded on the Cystic Fibrosis Mutation Database^5^. The novel variant was predicted to be deleterious by SIFT and the CADD (CADD PHRED score = 24.8). Located at the exon 11, the variant may affect the function of the nucleotide binding domains 1 (NBD1) domain after translation (Figure 3). Swiss model was also used to predict the protein tertiary structure, the amino acid change from valine to glutamic acid might affect the stability of the helix (Figure 4).

### Sanger Sequencing for Variant Validation of *CFTR*

**FIGURE 5:**
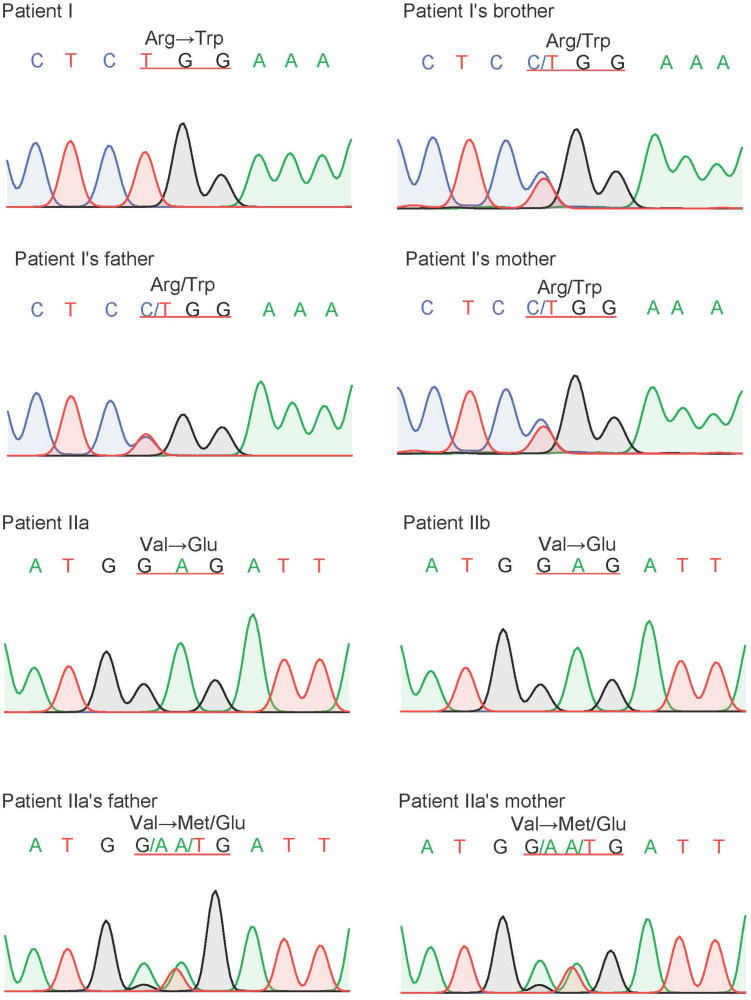
The result of Sanger sequencing of our probands and their family members. The variant of *CFTR*, c.1000C > T, p.R334W, were found in proband I and c.1409T > A, p.V470E were found in proband IIa and IIb. All of our probands are homozygous, and their parents are heterozygotes.

Sanger sequencing was used to validate the candidate variants identified by whole-exome sequencing, and segregation analyses were performed in both family members. Homozygous variant c.1000C > T, p.R334W in proband I and homozygous variant c.1409T > A, p.V470E in proband IIa and IIb were validated by Sanger sequencing. Sanger sequencing also confirmed that the healthy parents of the probands were all heterozygotes in corresponding positions (Figure 5).

## Discussion

In this study, whole-exome sequencing was performed to identify 2 variants in *CFTR* in 2 families. The parents of patients in both families are heterozygously married and are close relatives. A novel homozygous variant (c.1409T > A, p.V470E) was found in both patients in family II. It was located in the NBD1 domain of CFTR and may disturb the structure and function of ATP binding. Another reported homozygous variant (c.1000C > T, p.R334W) found in patient I was considered to be the cause of her respiratory disease.

**FIGURE 6:**
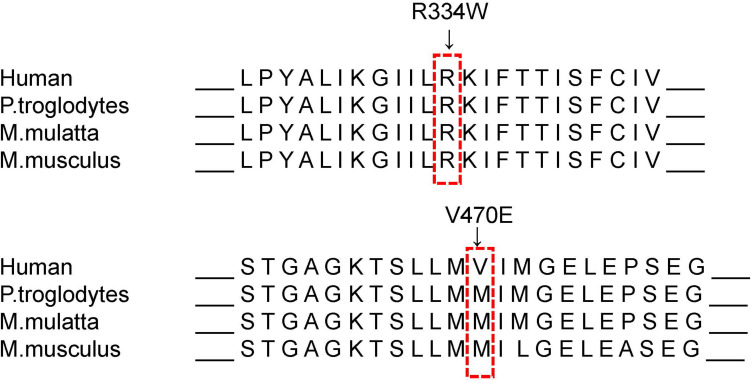
Sequence alignment of mammalian CFTR proteins. The R334 is located in a highly conserved amino acid, and the V470 is located at a variant position according to the MutationTaster.

The novel variant c.1409T > A, p.V470E in exon 11, which may affect the function of the NBD1 domain after translation, was not present in the current Cystic Fibrosis Mutation Database or the Human Gene Mutation Database^6^. Notably, this position is a polymorphic position of M470V (c.1408G > A) variants and is related to bronchiectasis and chronic pancreatitis by affecting structural stability of NBD1 and intrinsic chloride channel activity when it is combined with other variants (Lee et al., 2003; Yang et al., 2018). The V470 allele can reduce the activity of the CFTR protein chloride channel, which might help to reduce the disease damage and decrease the rate of diarrhea, primary sclerosing cholangitis, and prostate cancer (Henckaerts et al., 2009; Qiao et al., 2008). M470 allele is significantly associated with an increasing risk of chronic pancreatitis in both Asian and Caucasian populations (Zhou et al., 2020), which also contributes to a low birth rate through the congenital bilateral absence of the vas deferens (CBAVD) (Kosova et al., 2010). The variants in sequence among different races and species well reflect the natural selection during human evolution (Maria Ciminelli et al., 2011; Figure 6). The functional difference and species evolution caused by the polymorphic variants at this site also indicate that the variant V470E at this site has a high possibility of affecting the function of CFTR and causing disease. Both patients in family II have the typical cystic fibrosis clinical phenotype, and brother IIb, with no infection or symptoms, still had significant CT imaging findings of bronchiectasis and digital clubbing. This suggested a possible relationship between the variant and cystic fibrosis. According to ACMG guidelines, the novel variant was classified as the variant of uncertain significance (VUS), meeting the following criteria from the ACMG guidelines: PM2, PP1, PP3, and PP4 (Richards et al., 2015). Nevertheless, the phenotype-genotype association is hard to define completely because of the small sample size. *In vitro* validation is still in need for confirmation of this variant’s role in cystic fibrosis.

The variant found in proband I was a reported variant c.1000C > T, p.R334W, present in the common Caucasian *CFTR* mutation-screening panels (Gasparini et al., 1991). According to the database of CFTR2, there are 429 recorded data of alleles in this position, and only 40% of patients suffered from pancreatic insufficiency. Like most patients with this site variant, proband I showed no symptoms other than respiratory symptoms such as bronchiectasis and sinusitis. This variant is located in the eighth exon and the sixth transmembrane region, which is close to the gate situated between amino acid residues 337 and 344 (Beck et al., 2008; Gao and Hwang, 2015; Figure 4). Because it only affects the conductivity of the channel that decreases the flow of ions, the phenotype of patients is often comparably less severe. The variant was defined as a mild variant (Class IV) based on the pathogenic mechanism and severity of bronchus and pancreas (Ratjen et al., 2015).

The survival rate of CF patients has been greatly improved with the rapid development of treatments. The CFTR potentiator, ivacaftor, the first drug approved to be used in CF patients, is now approved to be used in many class III and class IV variants. Ivacaftor is also effective for the most common pathogenic variant, p.F508del, when combined with corrector lumacaftor. However, patient I in our study has the class IV variant R334W and is not a target of ivacaftor. New research showed that phosphodiesterase (PDE) inhibitors RPL554 (Verona Pharma) can elevate intracellular cAMP in R334W Fisher rat thyroid (FRT) cells. This finding supports the therapeutic potential of RPL554 for CF patients with class III/IV variants including R334W (Turner et al., 2020). In addition, gene and molecular therapy for some mutations is also a research hot spot. At present, the main difficulty is to find a suitable vector (Ratjen et al., 2015).

Cystic fibrosis is the most common and potentially fatal genetic disease in Caucasians, though it is rare in China. Here, we reviewed the 90 cases of Chinese CF patients with DNA sequencing (Supplementary Table 3). Like the previous review, only a few patients had pancreas insufficiency and CBVAD, and most patients have been diagnosed with bronchiectasis and lung infections (Shen et al., 2016). According to the results of a sputum culture, chronic infection of *Pseudomonas aeruginosa* was found in 62% of patients. Compared with the low prevalence of ABPA in CF patients in Europe, more than 25% of Chinese CF patients met the criteria for ABPA. Sweat testing has always been the gold standard for CF diagnosis; however, it has only been carried out in hospitals in some large cities. Only 66 of 90 cases had the data of sweat conductivity test. Different from the most common pathogenic variants p.F508del in Europe and America, c.2909G > A (p.G970D) was the most common pathogenic variant in China among all the variants. Research has shown that the *CFTR* variant spectrum of CF patients in China is different from that in Caucasians in Europe and America since some variants, such as c.1766 + 5G > T, only occur in Chinese people (Xu et al., 2017). It is necessary for us to establish a variant database of Chinese CF patients since most variants in Chinese CF patients have not been present in the common Caucasian *CFTR* mutation-screening panels. For patients with atypical clinical symptoms, it is difficult to make a clear diagnosis at an early stage without sweat tests or genetic testing, explaining the low diagnosis rate of CF in China. In the last decade, the number of reports of cystic fibrosis patients in China has increased significantly, and more cystic fibrosis patients and data will be discovered and reported in the future, after awareness and diagnosis techniques of the disease are improved.

## Conclusion

Our study used whole-exome sequencing to identify two homozygous variants of *CFTR*, including a novel variant (c.1409T > A, p.V470E) and a reported variant (c.1000C > T, p.R334W), in three individuals from two consanguineous families. The existence of the novel variant (c.1409T > A, p.V470E), which has never been reported, implies the association between the polymorphism of the site and the disease and indicates its changes in human evolution. In addition, our findings in the patient with variant c.1000C > T, p.R334W are the same as that of previous studies that showed that the phenotype identified by this variant is less severe. Although the current functional test and drug experiment for these variants have not achieved decisive results, we do think that they are necessary. Our review of Chinese CF patients also suggests that doctors should pay more attention to the atypical symptoms of Chinese CF patients. Our result expands the variant spectrum of *CFTR* and contributes to a more rapid genetic diagnosis and future genetic counseling.

## Data Availability Statement

The data that support the findings of this study are available from the corresponding author upon reasonable request.

## Ethics Statement

The studies involving human participants were reviewed and approved by Review Board of the Second Xiangya Hospital of Central South University in China. Written informed consent to participate in this study was provided by the participants’ legal guardian/next of kin. Written informed consent was obtained from the individual(s), and minor(s)’ legal guardian/next of kin, for the publication of any potentially identifiable images or data included in this article.

## Author Contributions

HL and ZT conceived and designed the experiments. BY, CL, and DY performed the experiments and analyzed the data. TG collected samples and the clinical data. BY and CL wrote the manuscript. All authors critically reviewed, revised, and approved the final version of the manuscript.

## Conflict of Interest

The authors declare that the research was conducted in the absence of any commercial or financial relationships that could be construed as a potential conflict of interest.

## Footnotes

1 https://cftr2.org

2 http://www.1000genomes.org

3 http://evs.gs.washington.edu/EVS/

4 https://gnomad.broadinstitute.org/

5 http://www.genet.sickkids.on.ca

6 http://www.hgmd.cf.ac.uk/ac/index.php
