# Supplementary material for: Whole-Exome Sequencing Identified CFTR Variants in Two Consanguineous Families in China
Source: Front Genet. 2021 Jul 2;12:631221. doi: 10.3389/fgene.2021.631221 (PMC8283821; doi:10.3389/fgene.2021.631221)
Supplement: Supplementary file 1 [file Data_Sheet_1.docx]

Supplementary Material

**Figure 1.**Whole-exome sequencing, variants filtering and validation procedure.


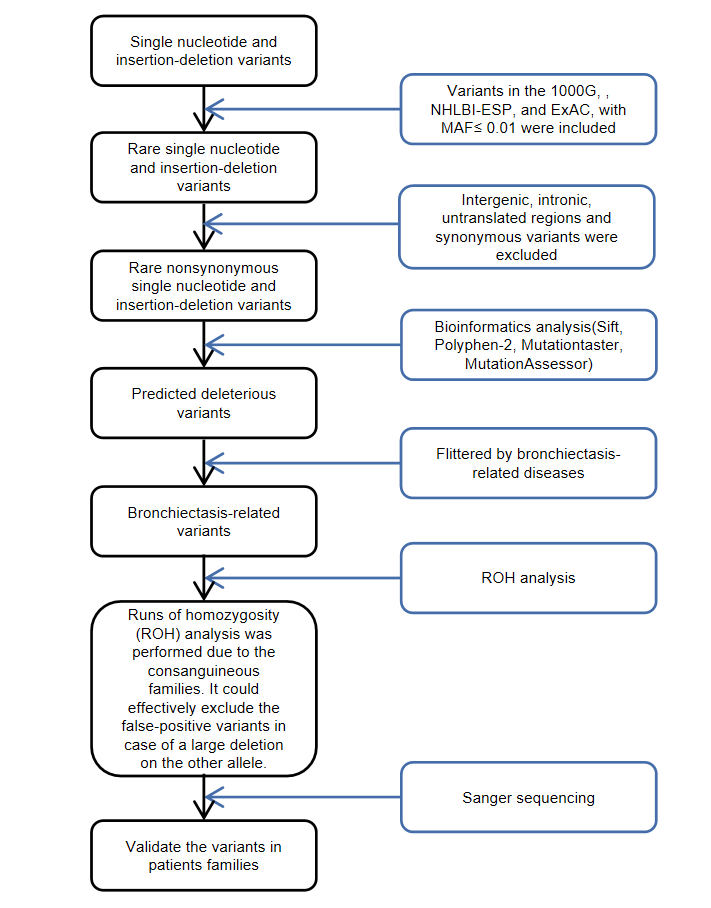


**Figure 2.** Homozygosity regions larger than 2 Mb are detected by AutoMap. Homozygosity regions are shown in blue and the region containing the *CFTR* gene is shown in red.


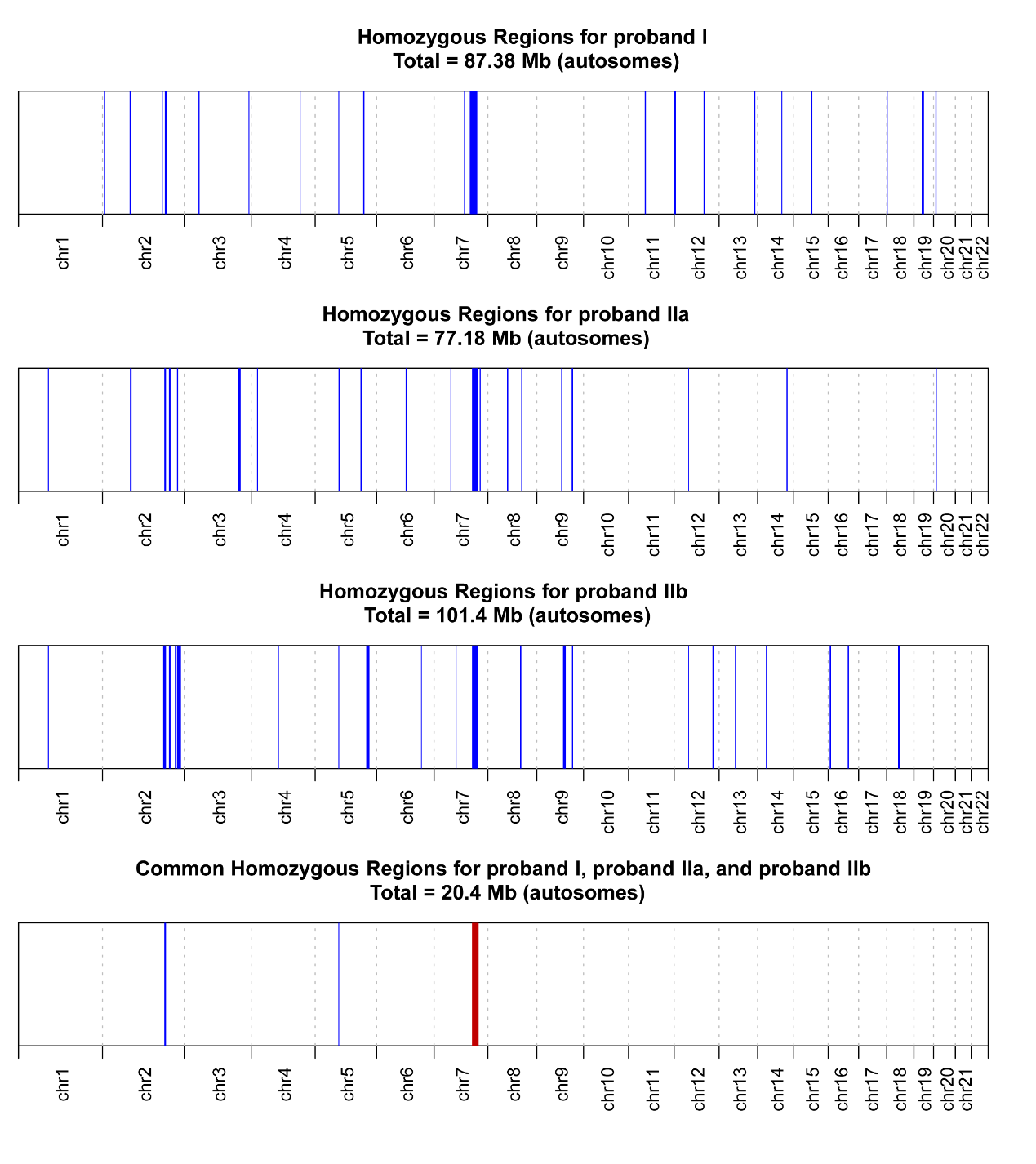


**Tables**

**Table 1.** Primers for PCR validation:

| Gene name | CDS | Primer F (5’-3’) | Primer R (5’-3’) | Product length |
| --- | --- | --- | --- | --- |
| *CFTR* | c.1000C>T p.R334W | AACTGAAACTGACTCGGAAGGC | TCTTCCTCCAAACCTATTCC | 229bp |
|  | c.1409T>A p.V470E | ATTGCTCCAAGAGAGTCATACCA | CTTTGATGACGCTTCTGTAT | 463bp |

**Table 2**. Summary of SNPs for exome captured samples.

|  | Patient Ⅰ | Patient Ⅱa | Patient Ⅱb |
| --- | --- | --- | --- |
| Exome Capture Statistics | | | |
| Raw data (G) | 14.24 | 7.09 | 7.25 |
| Total reads (Clean reads) | 93642380 | 46982228 | 47977086 |
| Total effective yields (Mb) | 13966.29 | 6989.48 | 7138.89 |
| Effective sequences on target (Mb) | 8719.72 | 4473.31 | 4655.45 |
| Fraction of effective bases on target | 62.4% | 64.0% | 65.2% |
| Average sequencing depth on target | 144.23 | 73.99 | 77.00 |
| Base covered on target | 60250047 | 60376237 | 60375307 |
| Coverage of target region | 99.7% | 99.9% | 99.9% |
| Fraction of target covered>=4× (%) | 99.6% | 99.7% | 99.7% |
| Fraction of target covered>=10× (%) | 99.4% | 99.0% | 99.1% |
| SNPs for exome capture | | | |
| Total number of SNVs | 156583 | 129696 | 129922 |
| Missense | 10149 | 10192 | 10170 |
| Nonsense | 71 | 72 | 77 |
| Splice site | 2483 | 2370 | 2348 |
| Synonymous-coding | 11283 | 11279 | 11303 |
| Total number of indels | 21242 | 15058 | 15433 |
| Frameshift_delection | 81 | 62 | 64 |
| Frameshift_insertion | 58 | 63 | 65 |
| Nonframeshift_delection | 205 | 188 | 196 |
| Nonframeshift_insertion | 197 | 173 | 178 |
| Stopgain | 8 | 4 | 3 |

**Table 3**.*CFTR* gene variants and clinical features in Chinese CF patients in published literatures.

| **No** | **Sex** | **Age at diagnose** | **cDNA change** | **Amino acid change** | **Clinical presentation** | **Sputum pathogens** | **Sweat conductivity(mmol/L)** |
| --- | --- | --- | --- | --- | --- | --- | --- |
| Ⅰ | F | 14 | c.1000C>T | p.R334W | Bronchiectasis/sinusitis | P aeruginosa | NA |
| Ⅱa | M | 17 | c.1409T>A | p.V470E | Bronchiectasis/sinusitis/recurrent pneumonia/finger clubbing | P aeruginosa | NA |
| Ⅱb | M | 22 | c.1409T>A | p.V470E | Bronchiectasis/sinusitis/finger clubbing | NA | NA |
| 1^[1]^ | F | 6 months | c.1766+5G>T | NA | Pneumonia/dehydration | NA | 127, 135 |
| 2^[2]^ | F | 5 | c.1766 + 1G>T | NA | Pneumonia/pancreatic insufficiency | NA | NA |
| 3^[3]^ | F | 8 | c.1766+5G>T | NA | Pneumonia/bronchiectasis/finger clubbing | P aeruginosa | NA |
| 4^[4]^ | F | 23 | c.2909G>A/c.319-326delGCTTCCTA | p.G970D/p.A107X | Bronchiectasis/sinusitis | P aeruginosa/S aureus/ B cepacia | 104 |
| 5^[5]^ | M | 17 | c.2083dupG/c.2684G>A/c.1766+5G>T | p.E695GfsX35/p.S895N | Bronchiectasis/recurrent pneumonia/finger clubbing | NA | 327 |
| 6^[5]^ | F | 14 | c.2083dupG/c.2684G>A/c.1766+5G>T | p.E695GfsX35/p.S895N | Bronchiectasis/recurrent pneumonia/finger clubbing | NA | 276 |
| 7^[6]^ | M | 3 | c.1657C>T | p.R553X | Bronchiectasis/recurrent pneumonia/pancreatic insufficiency/finger clubbing | P aeruginosa | NA |
| 8^[7]^ | F | 16 | c.567C>A/c.3691delT | p.N189K/p.S1231PfsX4 | Bronchiectasis/sinusitis/recurrent pneumonia/hemoptysis | P aeruginosa | 108.9 |
| 9^[8]^ | F | 12 | NA | p.W679X | Bronchiectasis/recurrent pneumonia/hemoptysis/pancreatic insufficiency | NA | 123.6 |
| 10^[9]^ | F | 13 | c.263T>G/c.2909G>A | p.L88X/p.G970D | Bronchiectasis/sinusitis/ABPA/hemoptysis | Aspergillus fumigatus | 98.53 |
| 11^[9]^ | F | 10 | c.3196C>T | p.R1066C | Bronchiectasis/sinusitis/pancreatic insufficiency/finger clubbing | P aeruginosa | NA |
| 12^[10]^ | F | 21 | c.293A>G | p.Q98R | ABPA/tuberculosis | Aspergillus fumigatus/tuberculosis | 154 |
| 13^[10]^ | M | 12 | c.95T>C/c.1657C>T | p.L32P/p.R553X | Bronchiectasis/ABPA/abdominal distension | Aspergillus fumigatus | 66 |
| 14^[10]^ | M | 10 | c.293A>G/c.558C>G | p.Q98R/p.N186K | ABPA/recurrent pneumonia | Aspergillus fumigatus | 135 |
| 15^[10]^ | M | 16 | c.2052 dupA/c.2909-?_3367+? del | p.Q686TfsX3/p.Gly980_Thr1112delinsGly | Bronchiectasis/recurrent pneumonia | NA | 132 |
| 16^[10]^ | F | 16 | c.2909G>A/c.744-?_1584+? del | p.G970D/p.Arg248_Glu528delinsArgfsX | Tuberculosis | Tuberculosis | 130 |
| 17^[10]^ | F | 28 | c.1666A>G | p.I556V | Tuberculosis/recurrent diarrhea | Tuberculosis | 100 |
| 18^[10]^ | F | 10 | c.1679+2T>C/c.2658-1G>C | NA | Bronchiectasis/ABPA | Aspergillus fumigatus | 154 |
| 19^[11]^ | M | 5 months | c.214G>A/c.650A>G/c.3406G>A | p.A72T/p.E217G/p.A1136T | Cholestasis jaundice | NA | NA |
| 20^[12]^ | F | 10 | c.595C>T | p.H199Y | Bronchiectasis/Sinusitis/Hepatocirrhosis/Pancreas disease | P aeruginosa | 306.8 |
| 21^[12]^ | M | 8 months | c.595C>T/c.2290C>T | p.H199Y/p.R764X | Bronchiectasis | Klebsiella pneumoniae/P aeruginosa | NA |
| 22^[13]^ | M | 11 | c.1699G>T/c.3909C>G | p.Asp567Tyr/p.Asn1303Lys | Bronchiectasis/sinusitis/ABPA/liver disease | Aspergillus fumigatus | 101 |
| 23^[13]^ | F | 10 | c.263T>G/c.1766+5G>T/c.*110C>G | p.Leu88X | Bronchiectasis/sinusitis/ABPA/ pancreatitis | S aureus | 103 |
| 24^[13]^ | M | 13 | c.3700A>G/c.960_961insA | p.Ile1234Val/p.Ser321IlefsX42 | Bronchiectasis/sinusitis/ABPA/hemoptysis/nasal polyps/failure to thrive | P aeruginosa | 101 |
| 25^[13]^ | F | 13 | c.263T>G/c.2909G>A | p.Leu88X/p.Gly970Asp | Bronchiectasis/Sinusitis/ABPA/Hemoptysis/clubbing finger | P aeruginosa | 99 |
| 26^[13]^ | M | 7 | c.326A>G/c.1000C>T/c.1666A>G | p.Tyr109Cys/p.Arg334Trp/p.Ile556Val | Bronchiectasis/Sinusitis/ABPA | Stenotrophomonas maltophilia | 106 |
| 27^[13]^ | F | 10 | c.595C>T | p.His199Tyr | Bronchiectasis/sinusitis/hepatocirrhosis/finger clubbing | P aeruginosa/S aureus | 127 |
| 28^[13]^ | F | 7 | c.223C>T/c.326A>G | p.Arg75X/p.Tyr109Cys | Bronchiectasis/sinusitis | P aeruginosa/A fumigatus | 118 |
| 29^[13]^ | F | 7 | c.1000C>T | p.Arg334Trp | Bronchiectasis/sinusitis | Klebsiella pneumoniae/S aureus | 118 |
| 30^[13]^ | F | 10 | c.263T>G | p.Leu88X | Bronchiectasis/recurrent pneumonia/failure to thrive/finger clubbing | P aeruginosa | 105 |
| 31^[13]^ | F | 11 | c.1666A>G | p.Ile556Va | Bronchiectasis/sinusitis/recurrent pneumonia | S pneumoniae | 96 |
| 32^[13]^ | M | 8 | c.293A>G/c.558C>G | p.Gln98Arg/p.Asn186Lys | Bronchiectasis/ABPA/recurrent pneumonia/failure to thrive | P aeruginosa | 115 |
| 33^[13]^ | F | 4 | c.326A>G/c.2374C>T | p.Tyr109Cys/p.Arg792X | Bronchiectasis/failure to thrive | P aeruginosa/S aureus | 101 |
| 34^[13]^ | M | 3 | c.1666A>G | p.Ile556Val | Sinusitis/recurrent pneumonia/bronchopulmonary dysplasia | S aureus | 99 |
| 35^[13]^ | F | 12 | c.293A>G | p.Gln98Arg | Bronchiectasis/sinusitis/ABPA/liver disease/finger clubbing | P aeruginosa | 122 |
| 36^[13]^ | M | 11 | c.648G>A/c.2491-126T>C | p.Trp216X | Bronchiectasis/sinusitis/recurrent pneumonia/hemoptysis/hepatocirrhosis/failure to thrive/finger clubbing/salty-tasting skin | P aeruginosa | NA |
| 37^[13]^ | F | 10 | c.3196C>T | p.Arg1066Cys | Bronchiectasis/sinusitis/recurrent pneumonia/failure to thrive/steatorrhea/atrophy of pancreas/finger clubbing/salty-tasting skin | P aeruginosa | NA |
| 38^[13]^ | M | 11 | c.414_415insCTA | p.Leu138_His139insLeu | Bronchiectasis/ABPA/liver disease/salty-tasting skin | P aeruginosa/S aureus/A fumigatus | NA |
| 39^[13]^ | F | 3 | c.1075C>T/c.3307delA | p.Gln359X/p.Ile1103X | Bronchiectasis/sinusitis/failure to thrive/steatorrhea/purulent appendicitis/finger clubbing/salty-tasting skin | P aeruginosa | NA |
| 40^[13]^ | F | 14 | c.2909G>A | p.Gly970Asp | Bronchiectasis/sinusitis/ABPA/failure to thrive/Steatorrhea/finger clubbing/salty-tasting skin | MRSA | 115 |
| 41^[14]^ | M | 20 | c.2909G>A/c.1521_1523delCTT | p.G970D/p.F508del | Bronchiectasis/Recurrent diarrhea | P aeruginosa | 137 |
| 42^[14]^ | F | 15 | c.2909G>A/c.2374C>T | p.G970D/p.R792X | Bronchiectasis | P aeruginosa | 140 |
| 43^[14]^ | F | 1 | c.2909G>A/c.2125C>T | p.G970D/p.R709X | Bartter Syndrome/Recurrent diarrhea/ Meconium ileus suspected | P aeruginosa/Staphylococcus Epidermidis | 108.4 |
| 44^[14]^ | M | 13 | c.3700A>G/c.959–960insA | p.I1234V/p.S321IfsX42 | Bartter Syndrome/Bronchiectasis/ABPA | P aeruginosa | 95.2 |
| 45^[14]^ | M | 15 | c.3635delT | p.V1212AfsX15 | Bronchiectasis | P aeruginosa | 106.5 |
| 46^[14]^ | F | 22 | c.2909G>A/c.1997T>G | p.G970D/p.L666X | Bronchiectasis/ABPA | P aeruginosa/Escherichia coli | 101.9 |
| 47^[14]^ | F | 4 | c.2909G>A/c.263T>G | p.G970D/p.L88X | Bronchiectasis/Malnutrition | P aeruginosa | 122.1 |
| 48^[14]^ | F | 13 | c.2909G>A/c.2907A>C | p.G970D/p.A969A | ABPA/Malnutrition | Negative | 62 |
| 49^[15]^ | M | 15 | c.865A>T/c.3651_3652 insAAAT | p.Arg289X/p.Tyr1219X | Bronchiectasis/finger clubbing/malnutrition/steatorrhea | NA | NA |
| 50^[15]^ | M | 12 | c.865A>T/c.3651_3652 insAAAT | p.Arg289X/p.Tyr1219X | Bronchiectasis | P aeruginosa | NA |
| 51^[16]^ | M | 5 | c.3196C>T/c.870-1G>C | p.R1066C | Bronchiectasis/nasosinusitis/ABPA/intestinal obstruction/pancreatic insufficiency | P aeruginosa | 140 |
| 52^[16]^ | F | 5 | c.3G>A/c.1572C>A | p.M1I/p.C524X | Bronchiectasis/Sinusitis/finger clubbing/ fat density of pancreatic | P aeruginosa | 109 |
| 53^[17]^ | M | 17 | c.1766+5G>T/c.3068T>G | p.I1023R | Bronchiectasis/recurrent pneumonia/respiratory failure | P aeruginosa | 121, 126 |
| 54^[17]^ | M | 6 months | c.1766+5G>T/c.3140-26A>G NA | - | Two episodes of pneumonia due to S aureus | S aureus | 100, 112 |
| 55^[17]^ | M | 2 months | c.868C>T/c.3068T>G | p.Q290X/p.I1023R | Bronchiectasis/segmental left lingular collapse | S aureus | 108, 112 |
| 56^[17]^ | F | 9 | c.1657C>T/c.3068T>G | p.R553X/p.I1023R | Bronchiectasis/Recurrent pneumonia/fatty change in liver | P aeruginosa | 123 |
| 57^[17]^ | F | 1 | c.3068T>G/c.3068T>G | p.I1023R | Bronchiectasis/sinusitis/recurrent pneumonia | P aeruginosa | 122, 124 |
| 58^[18]^ | M | 9 | c.579+1_579+2insACAT/c.1766+5G>T | - | Bronchiectasis/finger clubbing/hepatomegaly | P aeruginosa | NA |
| 59^[18]^ | M | 5 | c.595C>T | p.H199Y | Bronchiectasis/sinusitis/Malnutrition | P aeruginosa | NA |
| 60^[18]^ | F | 6 | c.1117-1G>C/c.2909G>A | p.G970D | Bronchiectasis/recurrent pneumonia | P aeruginosa | NA |
| 61^[18]^ | M | 13 | c.4056G>C | p.Q1352H | Steatorrhea/Bronchiectasis/Malnutrition | P aeruginosa | NA |
| 62^[19]^ | M | 4 | c.1657C>T | p.R553X | Bronchiectasis/sinusitis/fatty infiltration of the liver | P aeruginosa | NA |
| 63^[20]^ | F | 11 | c.3140-454_c.3367+249del931ins13 | NA | Bronchiectasis/otitis media | NA | 142 |
| 64^[21]^ | F | 6 months | c.532G>A | p.G178R | Bronchiectasis/Pseudo-Bartter syndrome | NA | NA |
| 65^[22]^ | F | 2 | c.753_754delAG/c.1240 C>T | p.Q414X | Bronchiectasis/sinusitis | P aeruginosa | NA |
| 66^[23]^ | M | 1.5 | c.19G>T, c. 860dupA | p.E7X/p.N287KfsX21 | Bronchiectasis/recurrent pneumonia/steatorrhea/developmental delay | NA | 89 |
| 67^[23]^ | F | 10 | c.1766+5G>T/c.2083dupG/c.2684G>A | p.E695GfsX35/p.S895N | Recurrent pneumonia/pancreatic insufficiency | NA | 135 |
| 68^[24]^ | F | 22 | c.2909G>A/c.3068 T> G | p.G970D/p.I1023R | Bronchiectasis/chronic gastritis/tuberculosis | P aeruginosa | 113.7 |
| 69^[24]^ | F | 11 | c.2909G>A/c.2997_  3000delAATT | p.G970D/p.I1000*  (3129del4) | Bronchiectasis/ABPA/diarrhea | P aeruginosa | 164.7 |
| 70^[24]^ | F | 14 | c.2909G>A/c.2909G>A | p.G970D/p.G970D | Bronchiectasis/ABPA/diarrhea/pancreatic insufficiency | MRSA/MSSA | 183 |
| 71^[24]^ | F | 18 | c.1766+5G>T/c.1766+5G >T | - | Bronchiectasis/ABPA/diarrhea | P aeruginosa/S aureus | 199 |
| 72^[24]^ | M | 22 | c.607A>T/c.3635delT | p.I203F/p.V1212Afs *16 | Bronchiectasis/Recurrent pneumonia | P aeruginosa | 151.4 |
| 73^[24]^ | F | 30 | c.2909G>A/c.2909G>A | p.G970D/p.G970D | Bronchiectasis/Sinusitis/hypoalbuminemia, | P aeruginosa | 117.9 |
| 74^[24]^ | F | 33 | c.2909G>A/c.2909G>A | p.G970D/p.G970D | Bronchiectasis | NA | 161.1 |
| 75^[24]^ | F | 4 | c.325T>G/c.3196C>T | p.Y109D/p.R1066C | fatty diarrhea/pancreatic insufficiency/sinusitis | P aeruginosa | 80 |
| 76^[24]^ | F | 18 | c.293A>G/c.2353C>T | p.Q98R/p.R785X | Bronchiectasis | P aeruginosa/MSSA | 85 |
| 77^[24]^ | F | 18 | c.54-?_273+?del(△E2–3) | - | diffusive pan-bronchiolitis/Sinusitis/hypoalbuminemia | P aeruginosa/MRSA | 120 |
| 78^[24]^ | F | 18 | c.3883_3886delATTT/c.2909G>A | p.I1295Ffs*32/p.G970D | Bronchiolitis/Sinusitis/hypoalbuminemia | Negative | 218.4 |
| 79^[24]^ | F | 16 | c.2909G>A/c.1657C> T | p.G970D/p.R553X | Bronchiectasis/Sinusitis/Nasal polyp/tuberculosis | P aeruginosa | NA |
| 80^[24]^ | M | 12 | c.405_406dupAC/c.1388G>A | p.L136Hfs*18/p.G463D | Bronchiolitis/Sinusitis/pancreatic insufficiency/hepatomegaly | P aeruginosa | 210 |
| 81^[24]^ | M | 22 | c.2125C>T/c.2909G>A | p.R709X/p.G970D | Bronchiectasis/Sinusitis/CBVAD/ | P aeruginosa/S aureus/Klebsiella pneumoniae | 192 |
| 82^[24]^ | M | 21 | c.2547C>A/c.2909G>A | p.Y849X/p.G970D | Bronchiectasis/ABPA/CBVAD/Sinusitis/pancreatic insufficiency/tuberculosis | P aeruginosa/MSSA | 133 |
| 83^[24]^ | F | 20 | c.3196C>A | p.R1066S | Bronchiectasis/Diffusive pan-bronchiolitis/Sinusitis/hypoalbuminemia | P aeruginosa | 63 |
| 84^[24]^ | F | 16 | c.1679+2 T>C/c.2658-1G>C | - | Bronchopneumonia/ABPA/sinusitis | P aeruginosa | 154 |
| 85^[24]^ | F | 22 | c.293A>G/c.293A>G | p.Q98R/p.Q98R | Bronchiectasis/ABPA | Negative | 81 |
| 86^[24]^ | M | 18 | c.595C>T/c.2909G>A | p.H199Y/p.G970D | Bronchiectasis/CBAVD/suspected pancreatic Insufficiency/abdominal distension | P aeruginosa | 99 |
| 87^[24]^ | F | 44 | c.1716C>A/c.2909G>A | p.D572E/p.G970D | Bronchiectasis | P aeruginosa | NA |

ABPA allergic bronchopulmonary aspergillosis, CBAVD congenital absence of the vas deferens, MRSA methicillin-resistant Staphylococcus aureus, MSSA methicillin sensitive Staphylococcus aureus, NA not available

**References**

[1] Wang MC, Shu SG, Chang SM, Ho WL, Chi CS. Cystic fibrosis in two Chinese infants in Taiwan. Zhonghua Min Guo Xiao Er Ke Yi Xue Hui Za Zhi. 1993. 34(4): 314-21.

[2] Crawford J, Labrinidis A, Carey WF, Nelson PV, Harvey JS, Morris CP. A splicing mutation (1898 + 1G-->T) in the CFTR gene causing cystic fibrosis. Hum Mutat. 1995. 5(1): 101-2.

[3] Zielenski J, Markiewicz D, Lin SP, Huang FY, Yang-Feng TL, Tsui LC. Skipping of exon 12 as a consequence of a point mutation (1898 + 5G-->T) in the cystic fibrosis transmembrane conductance regulator gene found in a consanguineous Chinese family. Clin Genet. 1995. 47(3): 125-32.

[4] Wagner JA, Vassilakis A, Yee K, et al. Two novel mutations in a cystic fibrosis patient of Chinese origin. Hum Genet. 1999. 104(6): 511.

[5] Wu CL, Shu SG, Zielenski J, Chiang CD, Tsui LC. Novel cystic fibrosis mutation (2215insG) in two adolescent Taiwanese siblings. J Formos Med Assoc. 2000. 99(7): 564-7.

[6] Chen H, Lin S, Lee H, et al. Cystic fibrosis with homozygous R553X mutation in a Taiwanese child. J Hum Genet. 2005. 50(12): 674.

[7] Li N, Pei P, Bu DF, He B, Wang GF. A novel CFTR mutation found in a Chinese patient with cystic fibrosis. Chin Med J (Engl). 2006. 119(2): 103-9.

[8] Cheng Y, Ning G, Song B, Guo YK, Li XS. A Chinese girl with cystic fibrosis: a case report identified by sweat and genetic tests. Chin Med J (Engl). 2012. 125(4): 719.

[9] Liu JR, Peng Y, Zhao YH, et al. [Clinical manifestations and gene analysis of 2 Chinese children with cystic fibrosis]. Zhonghua Er Ke Za Zhi. 2012. 50(11): 829-33.

[10] Liu Y, Wang L, Tian X, et al. Characterization of gene mutations and phenotypes of cystic fibrosis in Chinese patients. Respirology. 2015. 20(2): 312-8.

[11] Li L, Wang NL, Gong JY, Wang JS. [Infantile cholestasis caused by CFTR mutation: case report and literature review]. Zhonghua Er Ke Za Zhi. 2016. 54(11): 851-855.

[12] Xu BP, Wang H, Zhao YH, et al. [Molecular diagnosis of two Chinese cystic fibrosis children and literature review]. Zhonghua Er Ke Za Zhi. 2016. 54(5): 344-8.

[13] Shen Y, Liu J, Zhong L, et al. Clinical Phenotypes and Genotypic Spectrum of Cystic Fibrosis in Chinese Children. J Pediatr. 2016. 171: 269-76.e1.

[14] Tian X, Liu Y, Yang J, et al. p.G970D is the most frequent CFTR mutation in Chinese patients with cystic fibrosis. Hum Genome Var. 2016. 3: 15063.

[15] Xie Y, Huang X, Liang Y, et al. A new compound heterozygous CFTR mutation in a Chinese family with cystic fibrosis. Clin Respir J. 2017. 11(6): 696-702.

[16] Zheng B, Cao L. Differences in gene mutations between Chinese and Caucasian cystic fibrosis patients. Pediatr Pulmonol. 2017. 52(3): E11-E14.

[17] Leung GK, Ying D, Mak CC, et al. CFTR founder mutation causes protein trafficking defects in Chinese patients with cystic fibrosis. Mol Genet Genomic Med. 2017. 5(1): 40-49.

[18] Xu J, Yin Y, Zhang L, Zhang J, Yuan S, Zhang H. Four case reports of Chinese cystic fibrosis patients and literature review. Pediatr Pulmonol. 2017. 52(8): 1020-1028.

[19] Li H, Lin L, Hu X, Li C, Zhang H. Liver Failure in a Chinese Cystic Fibrosis Child With Homozygous R553X Mutation. Front Pediatr. 2019. 7: 36.

[20] Liu K, Liu Y, Li X, Xu KF, Tian X, Zhang X. A novel homozygous complex deletion in CFTR caused cystic fibrosis in a Chinese patient. Mol Genet Genomics. 2017. 292(5): 1083-1089.

[21] Yao Y, Feng XL, Xu BP, Shen KL. Pseudo-Bartter Syndrome in a Chinese Infant with Cystic Fibrosis Caused by c.532G>A Mutation in CFTR. Chin Med J (Engl). 2017. 130(22): 2771-2772.

[22] Wang YQ, Hao CL, Jiang WJ, et al. c.753_754delAG, a novel CFTR mutation found in a Chinese patient with cystic fibrosis: A case report and review of the literature. World J Clin Cases. 2019. 7(15): 2110-2119.

[23] Alper OM, Shu SG, Lee MH, et al. Detection of novel CFTR mutations in Taiwanese cystic fibrosis patients. J Formos Med Assoc. 2003. 102(5): 287-91.

[24] Liu K, Xu W, Xiao M, et al. Characterization of clinical and genetic spectrum of Chinese patients with cystic fibrosis. Orphanet J Rare Dis. 2020. 15(1): 150.
